# New Insights from the Oyster *Crassostrea rhizophorae* on Bivalve Circulating Hemocytes

**DOI:** 10.1371/journal.pone.0057384

**Published:** 2013-02-25

**Authors:** Mauro de Freitas Rebelo, Eliane de Souza Figueiredo, Rafael M. Mariante, Alberto Nóbrega, Cintia Monteiro de Barros, Silvana Allodi

**Affiliations:** 1 Instituto de Biofísica Carlos Chagas Filho, Universidade Federal do Rio de Janeiro, Rio de Janeiro, Rio de Janeiro, Brazil; 2 Instituto de Microbiologia Prof. Paulo de Góes, Universidade Federal do Rio de Janeiro, Rio de Janeiro, Rio de Janeiro, Brazil; 3 Núcleo em Pesquisa e Desenvolvimento Sócio-Ambiental de Macaé, Pólo Barreto, Campus Macaé, Universidade Federal do Rio de Janeiro, Macaé, Rio de Janeiro, Brazil; National Cancer Institute, United States of America

## Abstract

Hemocytes are the first line of defense of the immune system in invertebrates, but despite their important role and enormous potential for the study of gene-environment relationships, research has been impeded by a lack of consensus on their classification. Here we used flow cytometry combined with histological procedures, histochemical reactions and transmission electron microscopy to characterize the hemocytes from the oyster *Crassostrea rhizophorae*. Transmission electron microscopy revealed remarkable morphological characteristics, such as the presence of membranous cisternae in all mature cells, regardless of size and granulation. Some granular cells contained many cytoplasmic granules that communicated with each other through a network of channels, a feature never previously described for hemocytes. The positive reactions for esterase and acid phosphatase also indicated the presence of mature cells of all sizes and granule contents. Flow cytometry revealed a clear separation in complexity between agranular and granular populations, which could not be differentiated by size, with cells ranging from 2.5 to 25 µm. Based on this evidence we suggest that, at least in *C. rhizophorae*, the different subpopulations of hemocytes may in reality be different stages of one type of cell, which accumulates granules and loses complexity (with no reduction in size) as it degranulates in the event of an environmental challenge.

## Introduction

Hemocytes of bivalve mollusks are capable of phagocytosis, encapsulation, and enzymatic digestion [1], and play a major role in the immune system and homeostasis [2]. Their ability to transport proteins and substances such as heavy metals from organ to organ also confers on them an important role in response to toxins [3], [4]. This importance is not limited to beneficial roles, as hemocytes that have lost their ability to control cell division can give rise to a hemolymph neoplasia resembling human leukemia with a malignant phenotype [5]. Recent evidence from other invertebrates shows that hemocytes also have stem cell-like behavior, giving rise to neurons in the cerebral ganglia [6], [7], which suggests that these cells may be even more important than previously thought.

Despite their enormous potential for the study of physiological ecology, research on hemocytes has been impeded by the lack of a consensus on their classification. A plethora of categories has been established on the basis of different parameters and techniques [8]–[10], and as a consequence it is often difficult to compare results and draw general conclusions from the literature. The presence of granules in the cytoplasm has led several investigators to classify mollusk hemocytes as granulocytes and hyalinocytes (agranular) [11]–[13], a polarization that is clearly not sufficient to include all the variability observed in samples. On the other hand, the characterization based on cell differentiation and cell surface proteins [8], [9] has had the opposite effect, by adding too many categories. The lack of evidence on the origin of hemocytes contributes to the problem, since no correspondence between hemocyte subtypes and ontogeny can be made. Cell culture experiments that could help to sort out this problem are yet to come, because so far no stable cell line from invertebrates has been established. Today, all studies using bivalve hemocytes as models are still conducted with primary cultures. Recently, flow cytometry has been applied to analyze the variations in hemocyte number and frequency; however, the strength of the conclusions in these studies is, in our opinion, limited by the small number of specimens [10], [14], [15].

In this study, we approached this issue with a new protocol using flow cytometry combined with histological procedures, histochemical reactions, and transmission electron microscopy to characterize the hemocytes from the oyster *Crassostrea rhizophorae*. We interpret the resulting observations to mean that the so-called hemocyte subtypes are most likely different stages of a single cell type. This new insight can help to design future experiments using bivalve adults and hemocytes as models, and throw new light on the analysis of previous results.

## Materials and Methods

### Animals

Adult individuals of *C. rhizophorae* (5.2–9.1 cm long) were collected from aquaculture farms near the São João River in southeastern Brazil (22°48′S and 43°22′W), transported in local water in insulated plastic boxes, and maintained in aerated aquaria at a density of 1 oyster/L with reconstituted brackish water (dechlorinated tap water, salinity 10, pH 7.5) at 25°C for 7 to 15 days. The oysters were fed on spinach extract and *Saccharomyces cerevisae* until use. All procedures were conducted with appropriate concern for animal welfare.

### Hemolymph collection

Hemolymph from a total of 65 oysters was drawn from the adductor muscle with a 22-gauge needle fitted to a 5-mL syringe. For flow cytometry (from 25 oysters) the syringe contained 100 µL of a cold mixture containing 1 mL marine Alsever solution (MAS – 27 mM sodium citrate, 145 mM NaCl, 115 mM glucose, 9 mM EDTA in distilled water, pH 7.0) diluted in 3% paraformaldehyde [16], [17]. A volume of 900 µL of hemolymph was withdrawn with the syringe (completing 1 mL volume), and immediately after was transferred to an Eppendorf vial to complete the volume to 2 mL with the same solution. For both May-Grünwald-Giemsa staining (from 35 oysters) and histochemical reactions (from 5 oysters), hemolymph was simply drawn from the muscle and rapidly cytocentrifuged or dispensed onto coverslips to allow the hemocytes to adhere (see below).

### Light microscopy and histochemistry

For light microscopy, hemocytes were cytocentrifuged onto microscope slides and air-dried before being stained with May-Grünwald-Giemsa. The cells were characterized according to their morphological features.

For histochemical reactions, cells were allowed to adhere for 30 min onto coverslips previously coated with poly-L-lysine, fixed for 30 min in 4% paraformaldehyde diluted in artificial brackish water, and reacted to reveal acid phosphatase. In brief, they were incubated for 2 h at 37°C in a solution containing 32 mg β-sodium glycerophosphate and 20 mg lead citrate diluted in 10 mL 0.05 M sodium acetate buffer, pH 5.0. They were then rinsed with distilled water and incubated in a 1% ammonium sulfate solution. The slides were rinsed again and mounted with Entellan® (Merck). Because esterase is found in mature mammalian granulocytes, some coverslips were reacted for this enzyme using a Naphtol AS-D Chloroacetate Esterase Kit (Sigma # 91C-1KT) according to the manufacturer's instructions.

### Transmission electron microscopy

For transmission electron microscopy (TEM), the hemolymph from an additional 5 oysters and drawn from the adductor muscle was processed as described previously [18]. Briefly, they were harvested in MAS (pH 7.2) and centrifuged. Pellets were fixed for 2 h with 2.5% glutaraldehyde diluted in MAS, post-fixed for 45 min with 1% osmium tetroxide, dehydrated with acetone, and embedded in Polybed 812® resin. Ultrathin sections were obtained with an ultramicrotome (RMC-MT 6000-XL), mounted on copper grids, and then stained for 20 min with 2% uranyl acetate and 3 min with 1% lead citrate. The sections were analyzed by transmission electron microscope (Jeol JEM-1011) belonging to the Rudolf Barth Electron Microscopy Platform of the Oswaldo Cruz Institute/Fiocruz, and by transmission electron microscope (LM 906-Carl Zeiss) belonging to the Laboratório de Microscopia Eletrônica Prof. Luiz Henrique Monteiro Leal – LABMEL/Instituto de Biologia Roberto Alcântara Gomes/Universidade do Estado do Rio de Janeiro.

### Data acquisition and analysis by flow cytometry

Flow cytometric analyses were done in a FACSCalibur (BD Biosciences). Hemocytes collected from 25 oysters were stained with the nucleic acid stain SYBR® Green I (Invitrogen) in order to differentiate the cells from other particles in the hemolymph [19]. Gated SYBR® Green I positive cells were plotted in a side scatter (SSC) versus forward scatter (FSC) dot plot, and the different hemocyte subpopulations were analyzed and quantified. Acquisition was done with CellQuest (BD Biosciences), and FlowJo (Tree Star) was used for further analysis. Alternatively, hemocytes were sorted using a MoFlo Cell Sorter (Dako) equipped with the Summit V4.3 software, and each fraction collected was stained with May-Grünwald-Giemsa and analyzed by light microscopy.

### Differential cell counts

The different types of hemocytes were counted using light microscopy (routine-stained and histochemically reacted), TEM and flow cytometry. For light microscopy, hemocytes in two aliquots of 200 µL hemolymph stained with May-Grünwald-Giemsa were counted. At least 200 cells were counted on each slide, totaling 400 cells per oyster. Following the histochemical reactions, hemocytes in two additional aliquots of 200 µL hemolymph were counted. For TEM, 13 images were analyzed and a total of 78 cells were counted. For flow cytometry, cells were counted as described in the previous section.

### Statistical analysis

The differences between specific pairs of groups were tested for significance by Mann-Whitney non-parametric test. Significance was established at P<0.05 using GraphPad Prism version 5.01 (GraphPad Software, Inc.).

## Results

### Morphological and ultrastructural analyses of hemocytes

**Figure 1:**
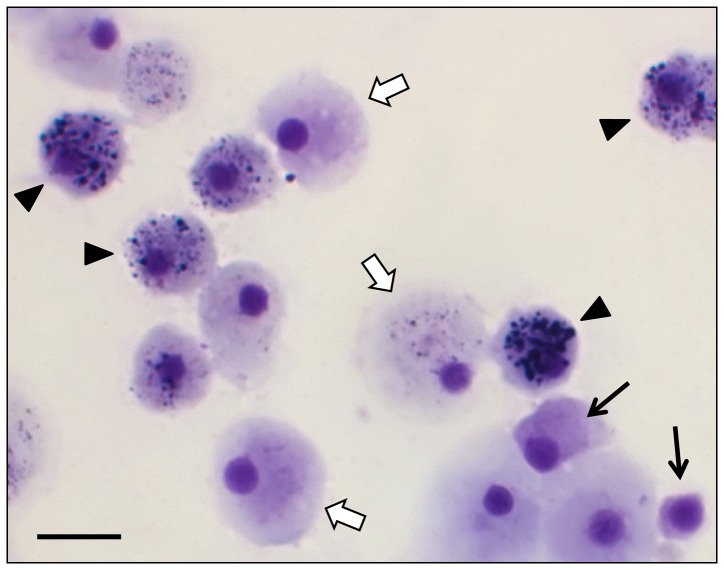
Light micrographs of fixed hemocytes of the bivalve *Crassostrea rhizophorae* stained with May-Grünwald-Giemsa. Hemoblast-like cells are indicated with narrow black arrows, agranular cells with large white arrows, and granular cells with black arrowheads. Note that some cells that are considered agranular may display a few small granular structures. Bar: 10 µm.

Light-microscopy observation of *C. rhizophorae* hemocytes stained with May-Grünwald-Giemsa showed three main cell types: hemoblast-like cells, agranular cells, and granular cells (Figure 1). Hemoblast-like cells were the smallest hemocytes, with a median diameter of 3.5 µm (min. 2.5 µm; max. 5 µm), spherical shape and high nucleus∶cytoplasm ratio. The agranular cells varied in diameter from 4 to 13 µm and were characterized by very few or no granules in the cytoplasm. Some agranular cells appeared round when attached to a glass slide, but others extended filopodia and spread over the slide. Granular cells had a median diameter of 15 µm (min. 11.5 µm; max. 18 µm), and were characterized by an eccentric nucleus and many cytoplasmic granules. Staining with May-Grünwald-Giemsa revealed that the hemoblast-like cells and agranular cells were slightly basophilic and that the granular cells had a basophilic cytoplasm, with basophilic and acidophilic granules (Figure 1).

**Figure 2:**
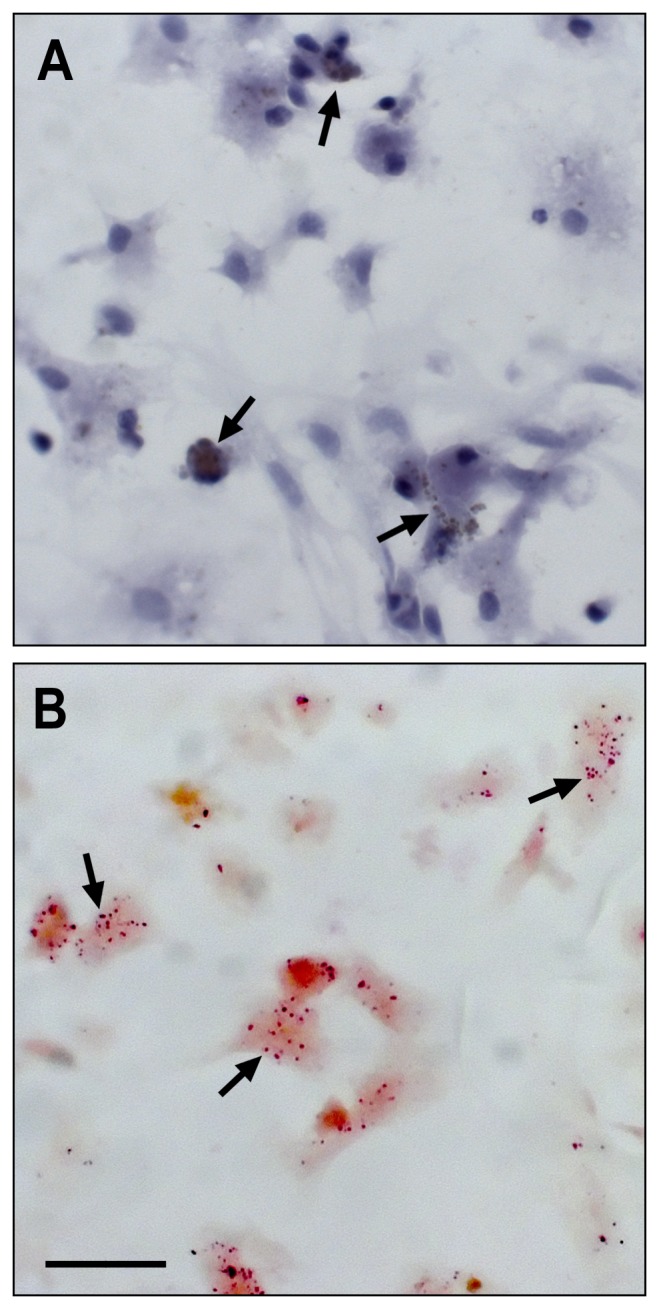
Histochemical reactions of hemocytes of *Crassostrea rhizophorae*. Acid phosphatase (A) and esterase (B) are within the granular cells. Black arrows indicate the granules reacted for each enzyme, respectively. Bar: 20 µm.

Esterase and acid phosphatase were revealed in granular cells by the specific histochemical reactions (Figure 2). A positive reaction for acid phosphatase indicated the presence of lysosomal granules in every cell type, with the exception of the hemoblast-like cells (Figure 2A). An esterase-positive reaction in several granular cells is shown in Figure 2B.

**Figure 3:**
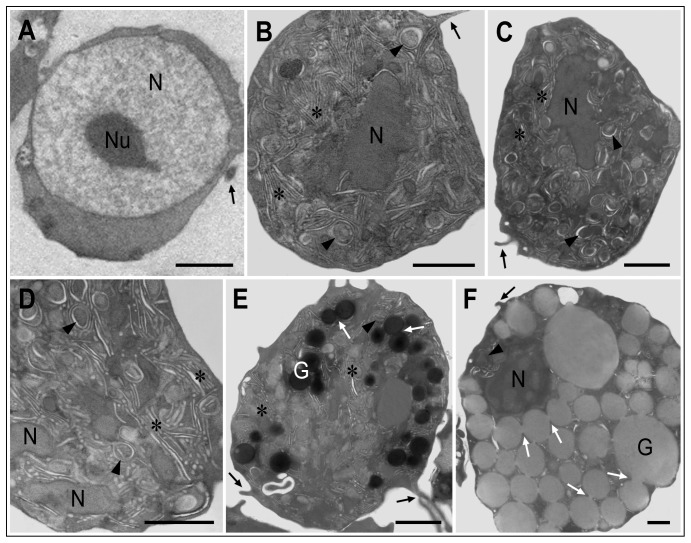
Transmission electron micrographs of hemocytes of *Crassostrea rhizophorae*. (A) Hemoblast-like cell displaying a very evident nucleolus (Nu) within the euchromatic nucleus (N). The cytoplasm shows homogeneously arranged dots, with a few membranous organelles. (B) A cell with many cisternae (asterisks) bounded by membranes, occupying most of the cytoplasm. Note that some of the cisternae have a core with variable electron densities (arrowhead). (C) Another hemocyte displaying cisternae in the cytoplasm, similar to that seen in (B). (D) Electron micrograph of the cytoplasm of a cell similar to those seen in (B) and (C) at higher magnification. Arrowheads indicate small granules surrounded by membranes continuous with the cisternae. (E) Hemocyte with several electron-dense granules (G). Some of the granules seem to be interconnected (white arrows). Note also electron-lucent granules within the cytoplasm, and cisternae (asterisks). (F) A granular cell with many large electron-lucent granules occupying almost the entire cytoplasm, and a few cisternae. Note that the granules are clearly interconnected to one another (white arrows). In (A–C), (E), (F) black arrows indicate finger-like cytoplasmic protrusions. Bars: 1 µm.

**Figure 4:**
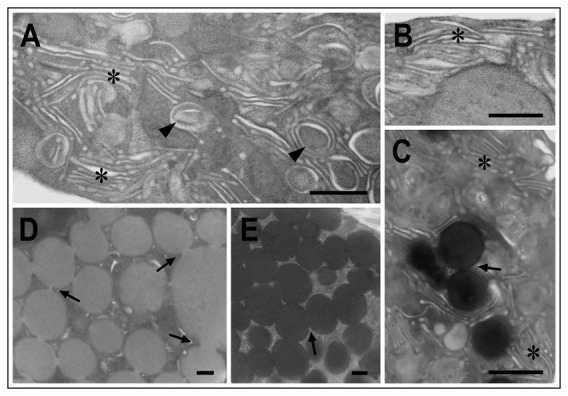
Electron micrographs showing cytoplasmic details of the hemocytes. (A) Cisternae (asterisks) and small granules surrounded by membranes (arrowheads) in the cytoplasm of cells similar to those shown in Figure 3B–D. (B) High magnification of cisternae (asterisk). (C) High magnification of the cytoplasm of a cell similar to that illustrated in Figure 3E, showing electron-dense granules surrounded by cisternae (asterisks). Note that some granules are interconnected (arrow). (D). Higher magnification of the cytoplasm of the hemocyte shown in Figure 3F, showing fused vesicles (arrows). (E) Cytoplasm of a hemocyte with many electron-dense granules. Note that the granules seem to be attached to one another. Bars: 500 nm.

Ultrastructural analyses by TEM revealed particular characteristics (Figure 3). In the hemoblast-like cell (Figure 3A), the most prominent feature was the presence of a nucleolus within a large euchromatic nucleus. The cytoplasm contained homogeneously distributed dots, with few, if any, membranous organelles. Figure 3B and C shows cells with many cisternae bounded by membranes, resembling the Golgi apparatus, possibly associated with the endoplasmic reticulum, and occupying most of the cytoplasm. Some of the cisternae contained material of different electron densities. Higher magnification of the cytoplasm of such a cell (Figure 3D) shows more detail of the cisternae and of granules in the process of formation. Figure 3E shows a cell displaying the same cisternae in the cytoplasm as those seen in Figure 3B to D; however, the cytoplasm of this cell contains several electron-dense lysosomes as well as electron-lucent granules. A granular cell with many large electron-lucent vesicles occupying almost the entire cytoplasm is shown in Figure 3F. Interestingly, here the vesicles are distinctly interconnected to one another, a feature that can also be seen in Figure 3E, and which strongly suggests fusion of vesicles for secretion by exocytosis (believed to be a function of the Golgi apparatus), as shown by [20]. Finger-like cytoplasmic extensions protruding from the cells can be seen in Figure 3 A to C, E and F. Figure 4 shows cytoplasmic details of the hemocytes described above. Golgi-like cisternae and membrane-bound vesicles in the cytoplasm of cells similar to those in Figure 3B to D are shown in Figure 4A, and the cisternae are shown enlarged in Figure 4B. A high-magnification electron micrograph (Figure 4C) shows the cytoplasm of a cell similar to that displayed in Figure 3E, containing lysosomes surrounded by cisternae. The interconnections of cell granules such as those displayed in Figure 3F are more clearly visible in Figure 4D. A few cisternae are also observed in this cell. Hemocytes with many electron-dense fused vesicles in the cytoplasm were also encountered (Figure 4E).

### Differential cell counts

A total of 5,720 routinely stained cells were counted under the light microscope: 71.06% of the cells were of the agranular type (median 3.5 µm; min. 2.5 µm; max. 5 µm), and 28.94% were granular (median 15 µm; min. 11.5 µm; max. 18 µm). Of the cells counted, 32% reacted for acid phosphatase, and 33% of the hemocytes reacted for esterase.

Of the 78 cells counted from the electron micrographs, 3.8% were hemoblast-like cells, similar to that seen in Figure 3A; 67.9% were agranular cells, similar to those in Figure 3B, C and D; 16.8% were granular cells with cisternae and a few electron-dense granules, as in Figure 3E; and 11.5% were granular cells with large granules, as in Figure 3F.

**Figure 5:**
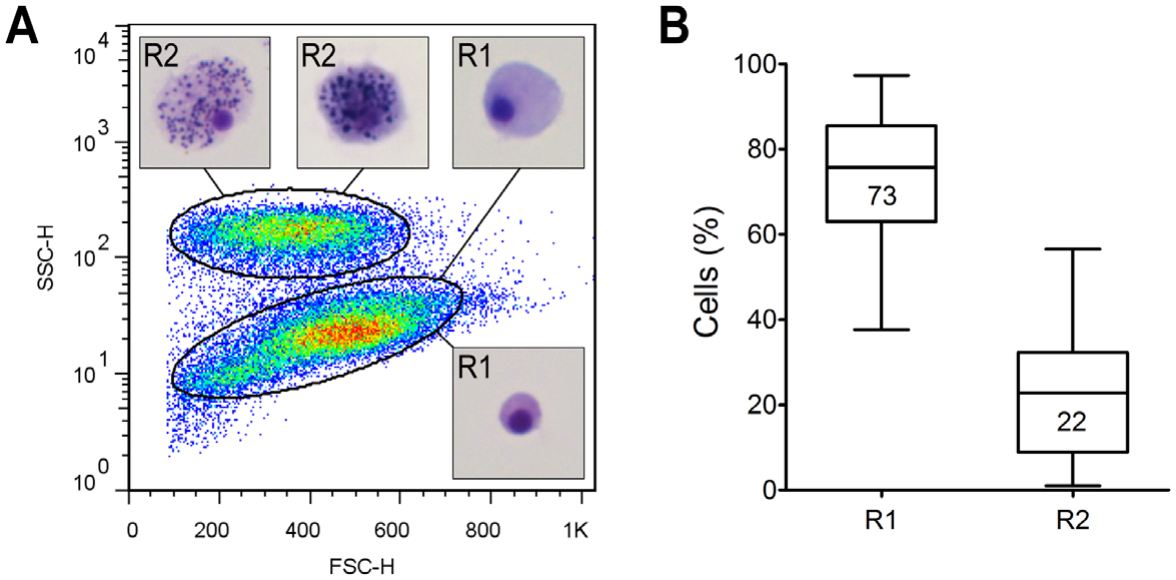
Representative flow-cytometry scatter plot of hemocytes from 25 oysters. Two populations designated R1 and R2, which comprise 73% and 22% of the total hemocytes, respectively, are shown (A, B). Inserts in (A) show representative cells from each population stained with May-Grünwald-Giemsa.

When the hemolymph from 25 oysters was analyzed by flow cytometry, two cell populations with distinguishable complexity (Side Scatter – SSC) and continuous size (Forward Scatter – FSC) were found (Figure 5A). A representative section R1 of the agranular population consisted of small- to medium-sized cells (med. 6.5 µm; min. 4 µm; max. 13 µm) with low to moderate granularity. However, cells in the agranular population could reach as much as twice this size. The representative section R2 of the high-granularity population consisted of medium to large cells (med. 10 µm; min. 5 µm; max. 25 µm). The agranular population comprised 73%, and the granular 22% of the total hemocytes (Figure 5B).

## Discussion

**Figure 6:**
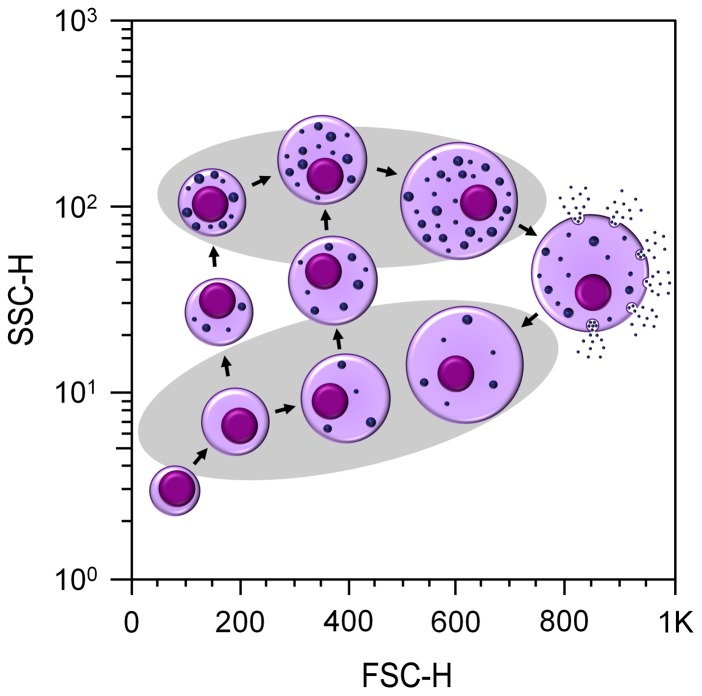
Proposed model for hemocyte maturation, as seen by flow cytometry. As hemoblasts mature they move toward the agranular population of hemocytes. As hemocytes increase in size they shift to the right, and as they accumulate granules they move up, frequently entering the highly granular population in the upper part of the plot. In the presence of an environmental challenge, granular hemocytes can degranulate, losing complexity and shifting back, down to the agranular population.

In this study, different approaches were used to examine the hypothesis that a single cell type could account for all the hemocyte morphologies that have been widely observed in bivalve hemolymph. The most widely accepted idea is that there are at least three types of hemocytes in bivalve mollusks [14], [21]–[23]. Our own initial observations using light microscopy also suggested this. However, the cell distribution profiles that we obtained with flow cytometry gave us a different perspective. The consistent two-cell population profile throughout the 25 samples that we analyzed could only be differentiated on the basis of complexity (or granule content - SSC), while the size (FSC) varied continuously over a wide range. Cells of different size classes could be observed in both the granular and agranular populations. To us, it was clear that rather than a snapshot of several cell populations with different size×complexity combinations that overlap with each other, we were looking at only one cell type that could be classified as younger, older, or post-reactive hemocytes, with or without granules. We propose the following developmental sequence: when hemoblast-like cells mature, they move into the agranular population that falls in the lower left part of the cytofluorograms. As these cells mature and their size increases they shift to the right, where the agranular population is, and up toward the population with more granules. Eventually, they migrate from the lower population to the highly granular population in the upper part of the scatter plot. Since granules develop slowly, some of these cells fall between the two populations in the cytofluorograms, as they move up. However, in the presence of an environmental or microbiological challenge, granular cells can degranulate, losing complexity and shifting back, down to the agranular population. Because degranulation does not reduce the cell size, large mature agranular cells accumulate on the right side of the scatter plot (Figure 6). In all our cytofluorograms, the continuous spread toward the right side of the dot plots clearly showed that at a certain time in the oyster's life, cells of all sizes and maturity stages are circulating in the hemolymph. This concords with the knowledge that invertebrate hemocytes barely divide and differentiate within the vessels. In decapod crustaceans, for example, the recovery after hemocytes have been eliminated by laminarin injection is mainly due to newly synthesized hemocytes in the hematopoietic tissue (20–30% proliferating cells) compared with the circulating hemolymph (1–2%) [24].

Electron-microscopy observations also contributed to this interpretation, as they revealed no specific subcellular differences between the cytoplasm of the recently differentiated hemocytes and that of more-mature cells. They all exhibited the cisternae that are stacked when the cells are small and become distended and filled with granules when they are large. Additional evidence came from histochemistry: both small and large granular cells reacted for acid phosphatase and esterase, but the hemoblast-like cells did not. These enzymes, which are typical of lysosomes and participate in the intracellular digestion of macromolecules and exogenous particles [25], [26], are widely accepted as markers of cell maturation. Similarly to the hemocytes of *C. rhizophorae* in our study, the histochemical reaction for esterase specifically evidenced granulocytes in late stages of maturation in a decapod crustacean [27], while the small hyalinocytes (hemoblasts) were the only cells with negative reactions for acid phosphatase and esterase in the clam *Tapes philippinarum* [28].

The granules seem, therefore, to constitute a fundamental aspect of the hemocyte morphology and physiology, and they are probably the factor underlying the extensive network of cisternae in the oyster cytoplasm. It has been shown that granules, including in oyster hemocytes, may be rich in antimicrobial peptides that are bound selectively to phagosomes, or are rapidly released in the circulation in the event of environmental demand [29]–[31]. A voluminous Golgi apparatus associated with the endoplasmic reticulum would allow rapid phagocytosis and exocytosis, as well as the transport addressing of large amounts of peptides to vesicles after post-transcriptional modifications. The cisternae unfold during maturation to surround the granules, and then continue to communicate as the cells degranulate. This feature has been previously described in the hemocytes of the oyster *Crassostrea virginica* after a challenge [32] and displayed by TEM in experimentally stimulated cells, such as vertebrate mast cells or pancreatic acinar cells [33]. This extensive network of membrane cisternae, which resembles the Golgi apparatus (possibly associated with the endoplasmic reticulum), is an unusual feature that was observed here for the first time in bivalve hemocytes.

The limitations of hemocyte counts and observations with light microscopy may have led to a ‘quest’ for hemocyte types with different origins, which, in our opinion, do not exist: the categories were an artifact of the small number of replicates. If we examine different hemocyte types listed in the literature [13], although they are supported by observations, there is not a unifying model that could explain the different types observed. In *Mytilus edulis*, for example, monoclonal antibodies specific for hemocyte subpopulations were generated using separate basophilic and eosinophilic cell types as antigens, but they clearly reacted with both the subpopulations of granular hemocytes and hyaline cells [8]. The same holds true for the oyster *Ostrea edulis*. Five out of six monoclonal antibodies developed for hemocytes showed specificity for more than one hemocyte type [34].

To conclude, we found no evidence of more than one origin for mature hemocytes in the oyster *C. rhizophorae*. Based on three different approaches that revealed biochemical, morphological, and ultrastructural characteristics common to cells of all sizes, we feel confident in stating that the variety of hemocytes seen in a snapshot of the hemolymph at a given time is the result of different stages of maturation and function of the same cell type. These cells are able to form granules (most likely rich in antimicrobial substances) and degranulate in response to challenges, in a model that is more suggestive of a single origin than of a plethora of hemocyte categories.
